# Consistent genes associated with structural changes in clinical Alzheimer’s disease spectrum

**DOI:** 10.3389/fnins.2024.1376288

**Published:** 2024-11-01

**Authors:** Yingqi Lu, Xiaodong Zhang, Liyu Hu, Qinxiu Cheng, Zhewei Zhang, Haoran Zhang, Zhuoran Xie, Yiheng Gao, Dezhi Cao, Shangjie Chen, Jinping Xu

**Affiliations:** ^1^Department of Rehabilitation Medicine, The People’s Hospital of Baoan Shenzhen, Shenzhen, China; ^2^Department of Rehabilitation Medicine, The Second Affiliated Hospital of Shenzhen University, Shenzhen, China; ^3^Institute of Biomedical and Health Engineering, Shenzhen Institutes of Advanced Technology, Chinese Academy of Sciences, Shenzhen, China; ^4^Shenzhen Children’s Hospital, Shenzhen, China

**Keywords:** Alzheimer’s disease, mild cognitive impairments, gray matter volume, gene expression, enrichment analysis

## Abstract

**Background:**

Previous studies have demonstrated widespread brain neurodegeneration in Alzheimer’s disease (AD). However, the neurobiological and pathogenic substrates underlying this structural atrophy across the AD spectrum remain largely understood.

**Methods:**

In this study, we obtained structural MRI data from ADNI datasets, including 83 participants with early-stage cognitive impairments (EMCI), 83 with late-stage mild cognitive impairments (LMCI), 83 with AD, and 83 with normal controls (NC). Our goal was to explore structural atrophy across the full clinical AD spectrum and investigate the genetic mechanism using gene expression data from the Allen Human Brain Atlas.

**Results:**

As a result, we identified significant volume atrophy in the left thalamus, left cerebellum, and bilateral middle frontal gyrus across the AD spectrum. These structural changes were positively associated with the expression levels of genes such as ABCA7, SORCS1, SORL1, PILRA, PFDN1, PLXNA4, TRIP4, and CD2AP, while they were negatively associated with the expression levels of genes such as CD33, PLCG2, APOE, and ECHDC3 across the clinical AD spectrum. Further gene enrichment analyses revealed that the positively associated genes were mainly involved in the positive regulation of cellular protein localization and the negative regulation of cellular component organization, whereas the negatively associated genes were mainly involved in the positive regulation of iron transport.

**Conclusion:**

Overall, these results provide a deeper understanding of the biological mechanisms underlying structural changes in prodromal and clinical AD.

## 1 Introduction

Mild cognitive impairment (MCI), a transitional and intermediate state between normal aging and Alzheimer’s disease (AD), however, may have a significantly higher risk of converting to probable AD than the normal population. The conversion rate of MCI patients to AD was at an average of 10–17% per year (Petersen et al., 2009; Landau et al., 2010; Davatzikos et al., 2011) and approximately 60% within 10 years (Mitchell and Shiri-Feshki, 2009). A recent follow-up study even reported that the majority (45.5%) of those MCI individuals subsequently developed AD for an average of 26.6 months (Espinosa et al., 2013). The high risks make it highly important to involve the early prodromal stage, especially MCI, in exploring neurobiological and pathogenic substrates of AD.

Gray matter volume (GMV) atrophy is one of the main cardinal signs of neurodegeneration in AD and is irreversible. There is a long preclinical stage of AD, in which no obvious symptoms but subtle structural changes in specific brain regions can be detected (Tondelli et al., 2012). In the early stage (MCI), marked localized atrophy could occur in many cortical regions and certain sub-cortical regions. During the progression from MCI to AD, global and local GMV atrophy was reported mainly in the temporal neocortex, parahippocampal cortex, and cingulate gyrus (Spulber et al., 2012). Subsequently, this atrophy spreads aggressively to affect most of the brain in clinical AD (Pini et al., 2016). Although the neurobiological and pathogenic substrates underlying particular structural changes across AD spectrum have been investigated using MRI-based genetic study, such as the associations between genetic variations within PARP1 and CARD10 and a more rapid rate of hippocampal volume loss (Nho et al., 2013a,b), between the TREM2 variant and fronto-basal gray matter loss (Luis et al., 2014), between APOE and longitudinal change in the hippocampus (Apostolova et al., 2014; Andrawis et al., 2012), between expression level of ABCA7 and GMV changes in post-central gyrus, between superior frontal gyrus and ZCWPW1, and between right post-central gyrus and APOE (Roshchupkin et al., 2016) were identified, much more AD risk variants have been reported (Guo et al., 2017; Lacour et al., 2017; Shen and Jia, 2016). For example, a large meta-analysis on GWAS involved 74,046 people and identified 20 risk genes and 11 new susceptibility loci associated with AD (Lambert et al., 2013). Since a fraction of MCI patients are in the pre-AD stages, AD risk alleles, as well as additional genetic factors specifically influencing MCI progression, have received extensive attention (Moreno-Grau and Ruiz, 2016). Indeed, some major pathogenic genes were identified in MCI using open gene expression data sets (Tao et al., 2020). Recently, a meta-analysis also revealed several abnormally regulated genes, shared pathways, and transcription factors in MCI and AD (Bottero and Potashkin, 2019). However, one previous study identified that gene expression patterns in MCI are neither an extension of aging nor an intermediate between aged controls and AD (Berchtold et al., 2014). In the Chinese population, the SORL1 genetic variants, especially polymorphism rs985421, were identified to reduce the risk of converting from MCI to AD (Gao et al., 2014; Jin et al., 2014). Considering this evidence, it is really important to investigate whether structural changes in the AD spectrum were driven by similar gene variants.

Recently, the Allen Human Brain Atlas (AHBA^1^) microarray dataset provided an indirect way for relating brain-wide transcriptomic data to neuroimaging data (Arnatkeviciute et al., 2019). The practical pipeline has been verified in various brain disorders, such as major depressive disorders (Anderson et al., 2020; Tan et al., 2021; Ji et al., 2021), schizophrenia (Liu et al., 2019), and migraine (Eising et al., 2016). Moreover, the expression level of genes involved in mitochondrial respiration and metabolism of proteins was found to be associated with regional GMV patterns across AD-memory, AD-executive, AD language, and AD-visuospatial subgroups (Groot et al., 2021). Therefore, it is ideal to investigate the relationship between transcriptional data and GMV changes in prodromal and clinical AD using this method, which will open up a new view to advance our understanding of the biological mechanisms underlying structural changes in AD.

**Figure 1:**
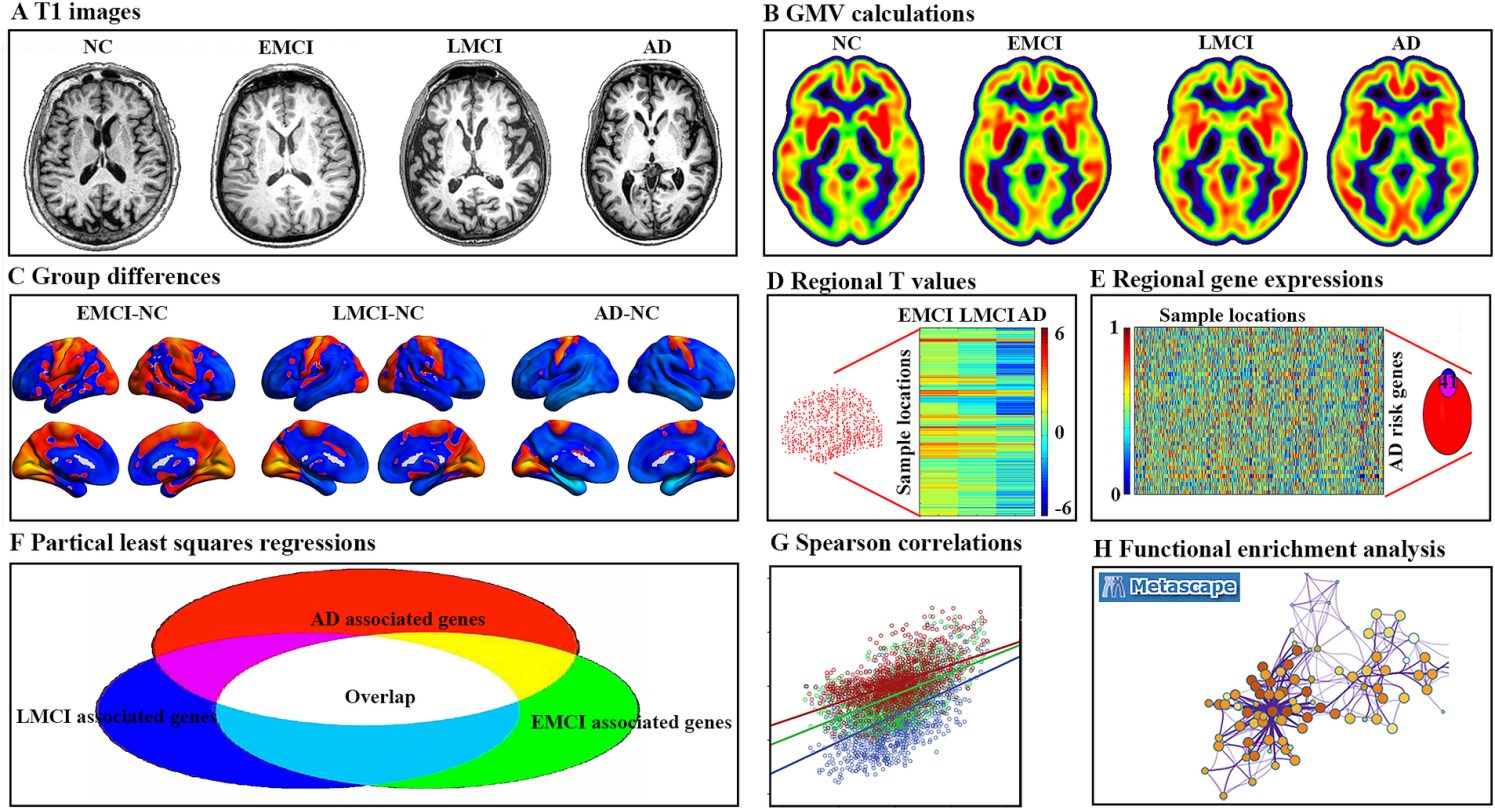
Pipeline of data processing. (A) Example T1 image for normal controls (NC), early mild cognitive impairment (EMCI), late mild cognitive impairment (LMCI), and Alzheimer’s disease (AD). (B) Gray matter volume (GMV) for each example. (C) Two sample t-tests were used to obtain voxel-wise GMV differences between EMCI, LMCI, AD, and NC. (D) Regional *T* value for each tissue sample in the left hemisphere. (E) Gene expression values of AD risk genes in tissue samples were obtained in six donated brains from the Allen Human Brain Atlas. (F) Gene-wise cross-sample partial least squares (PLS) regressions were performed between gene expression and GMV differences, respectively. The intersected genes were defined as genes associated with GMV alterations for all three groups. (G) Spearman correlations between gene expression levels of overlap genes and regional GMV difference. (H) Functional enrichment analysis using Metascape.

In the current study, major steps were performed according to the schematic summary of the processing pipeline (Figure 1): (1) GMV changes were analyzed using two-sample t-tests for each patient’s group compared to NC; (2) gene expression levels were obtained from the AHBA data and processed using the new pipeline; (3) regional GMV changes and regional gene expression level for interesting genes were calculated for each sample locations; (4) cross-sample spatial correlations between gene expression levels and GMV alterations were performed using partial least square regression (PLS) for each group; (5) obtain consistent genes among three group; (6) Spearman correlations between gene expression levels of consistent genes and GMV changes were performed for each group; and (7) functional enrichment analysis was conducted using Metascape analysis^2^ to explore ontological pathways of the consistent genes.

## 2 Materials and methods

### 2.1 Participates

To ensure consistency, the Alzheimer’s Disease Neuroimaging Initiative (ADNI) database^3^ was searched for normal controls (NC), early mild cognitive impairment (EMCI), late mild cognitive impairment (LMCI), and AD who were imaged at baseline using a 3 Tesla MRI scanner. Subjects were excluded if they did not have a Mini-Mental State Examination (MMSE) score or failed image registration or segmentation. As a result, a total of 514 subjects (83 NC, 217 EMCI, 120 LMCI, and 94 AD) were remained. Since the number of subjects varies very much across four groups, sub-groups of 83 EMCI, 83 LMCI, and 83 AD were randomly chosen to match the age and gender of 83 NC and were further used in the current study (Table 1). Specifically, EMCI and LMCI patients were all amnestic and were diagnosed based on the following criteria: (1) a subjective memory concern reported by themselves, their partner, or a clinician; (2) MMSE score between 24 and 30; (3) Clinical Dementia Rating (CDR) of 0.5 in the memory box; (4) cognitive and functional performance was not sufficient to diagnose as AD on the screening visit; and (5) scored 9–11 with 16 or more years of education, 5–9 for 8–15 years of education, or 3–6 for 0–7 years of education on the logical memory II subscale of the Wechsler Memory Scale-Revised for EMCI, whereas scored less than or equal to 8 for 16 or more years of education, less than or equal to 4 for 8–15 years of education, or less than or equal to 2 for 0–7 years of education for LMCI. All AD patients had to meet the criteria for probable AD according to the NINCDS-ADRDA criteria, and detailed information can be referred to the ADNI manual.^4^

**Table 1:** Data characteristics.

Clinical characteristics	AD	LMCI	EMCI	NC	*p* value^a^
Subjects	83	83	83	83	–
Age (mean ± SD)	75.39 ± 7.16	75.20 ± 6.3	76.06 ± 5.91	75.92 ± 5.73	0.787
Gender (male/female)	39/44	38/45	38/45	38/45	0.998
MMSE scores	21.49 ± 3.52	26.81 ± 2.56	27.79 ± 1.57	29.24 ± 0.89	<0.001

AD, Alzheimer’s disease; LMCI, late mild cognitive impairment; EMCI, early mild cognitive impairment; NC, normal controls; SD, standard deviation; MMSE, mini-mental state examination. PLS1 scores and Spearman correlations between gene expression levels of overlap genes and regional GMV difference of cEMCI, sEMCI, cLMCI, and sLMCI patients compared to NC.

a Represents a one-way analysis of variance (ANOVA).

### 2.2 MRI data acquisition

Raw, unprocessed 3.0 T T1-weighted MRI images were downloaded from the ADNI database and scanned using different MRI scanners at multiple sites. Details about the data acquisition protocol can be seen on ADNI’s official webpage.^5^

### 2.3 Data preprocessing

The T1 images were preprocessed using the standard pipeline in the DPABI toolbox^6^ with unified segmentation and diffeomorphic anatomical registration through the exponentiated lie algebra (DARTEL). The major steps were: (1) segmenting each image into gray matter, white matter, and cerebrospinal fluid; (2) normalization using the DARTEL; (3) resampling to a voxel size of 1.5 mm × 1.5 mm × 1.5 mm; (4) modulating by multiplying the voxel values with the Jacobian determinant derived from the spatial normalization; and (5) smoothing with a Gaussian kernel of 8 mm × 8 mm × 8 mm full width at half maximum.

### 2.4 Gene expression data processing

We processed gene expression data of six postmortem adult brains using a new pipeline^7^ (Arnatkeviciute et al., 2019), which contains gene expression measurements from six adult donor brains. These brains were meticulously dissected using either manual or laser methods, resulting in a total of 3,702 sample sites in various regions, including the cerebral cortex, subcortical areas, cerebellum, and brainstem. Here, two of the donor brains provided samples from both hemispheres, while the remaining four brains were sampled from the left hemisphere only. To control for variability in gene expression between hemispheres, our analysis focused only on left-hemisphere samples from all donor brains. The major steps were: (1) reassigning probes to genes to the latest version using the Re-annotator toolkit^8^; (2) based on the binary indicators provided by AHBA, we selected probes that showed signal above background noise in at least 50% of sample sites; (3) since different probes measuring same gene may yield different expression levels due to different probe sensitivities, we ensured the robustness of gene expression measurements by selecting probes corresponding to genes present in both microarray and RNA-seq datasets, and chose the probe measurements that were most highly correlated with the RNA-seq values to represent gene expression; and (4) normalize within and between brains to minimize non-biological bias while preserving biologically relevant differences. Ultimately, our analysis encompassed 1,285 samples, with each tissue sample covering up to 10,027 genes.

### 2.5 Regional GMV differences

To explore the overall difference among Alzheimer’s spectrum, the voxel-wise GMV differences among EMCI, LMCI, AD, and NC were performed using multivariate analysis of variance (MANOVA) and post-hoc analyses between any other two groups. As we aimed to investigate voxel-wise GMV differences map for patients at different stages (EMCI, LMCI, and AD) as compared to matched NC, we also performed three two-sample *t*-tests (EMCI-NC, LMCI-NC, and AD-NC), respectively. At the same time, two-sample t-tests were performed to compare differences between different stages (EMCI vs. LMCI, EMCI vs. AD, LMCI and AD). All these results were corrected using a Gaussian random field (GRF, a cluster level of *p* < 0.05, and a voxel level of *p* < 0.001). Negative and positive overlap among the three groups was obtained using the intersection.

Moreover, spheres with a radius of 4.5 mm (i.e., three times the voxel size) centered in the MNI coordinate of each tissue sample (*n* = 1,285) were drawn, and the regional mean *T*-value within this sphere was defined as the t-statistic value of GMV difference for three groups, respectively.

### 2.6 AD risk genes associated with GMV differences

Fifty-two reproducible and established AD risk genes based on recently published literature (Lancour et al., 2020) were intersected with 10,027 background genes, resulting in 41 interesting genes. Then, we calculated a matrix of 1,285 regions × 41 gene expressions. To further explore their relationship with the GMV difference, partial least squares (PLS) regression was performed with gene expression data as predictor variables (Abdi and Williams, 2013). The first component of the PLS (PLS1) was further used in the current study, which was the linear combination of gene expression values that was most strongly correlated with regional changes in GMV difference. Then, cross-sample non-parametric Spearman rank was performed to determine the relationship between regional PLS1 weighted gene expression and regional GMV alterations. To estimate the variability of the PLS1 score for each gene, bootstrapping 1,000 times was performed. Z scores were defined as the ratio of the weight of each gene to its bootstrap standard error, and the genes were ranked according to their contributions to PLS1 using univariate one-sample Z tests (Morgan et al., 2019). The set of genes with *Z* > 5 or *Z* < −5 were considered as positive or negative associated gene lists. All these above steps were performed to correct multiple comparisons. This procedure was performed separately for each dataset. The final gene sets were defined as the overlap between the two datasets (interaction).

### 2.7 Analyses for consistent genes

Cross-sample non-parametric Spearman correlations were performed to explore the relationship between gene expression level and GMV changes (T values) for 1,285 regions in each group. Moreover, the number of comparisons (*n* = 12) was further corrected with a significance threshold of *p* < 4.16 × 10–3 = 0.05/12 (Bonferroni correction).

### 2.8 Re-analyses for sub-groups

Since EMCI and LMCI consist of patients who were ultimately converted to AD, remitted to NC, and stable in MCI, we subdivided them into convert (cEMCI and cLMCI), stable (sEMCI and sLMCI), and remitted sub-groups. The remitted sub-groups were relatively small and were not included in the further analyses. The four other sub-groups (15 cEMCI, 59 sEMCI, 43 cLMCI, and 35 sLMCI) were analyzed using the same method. The weighted PLS1 scores and r values of Spearman correlation were calculated for each group.

### 2.9 Functional enrichment analyses

To understand pathways of gene ontology (GO) biological processes, molecular functions, cellular components, and Kyoto Encyclopedia of Genes and Genomes (KEGG) pathways, we performed Metascape analysis (Zhou et al., 2019) using the positive and negative associated gene lists, respectively. The obtained enrichment pathways were thresholded for significance at 5% with at least three genes.

## 3 Results

### 3.1 GMV differences

**Figure 2:**
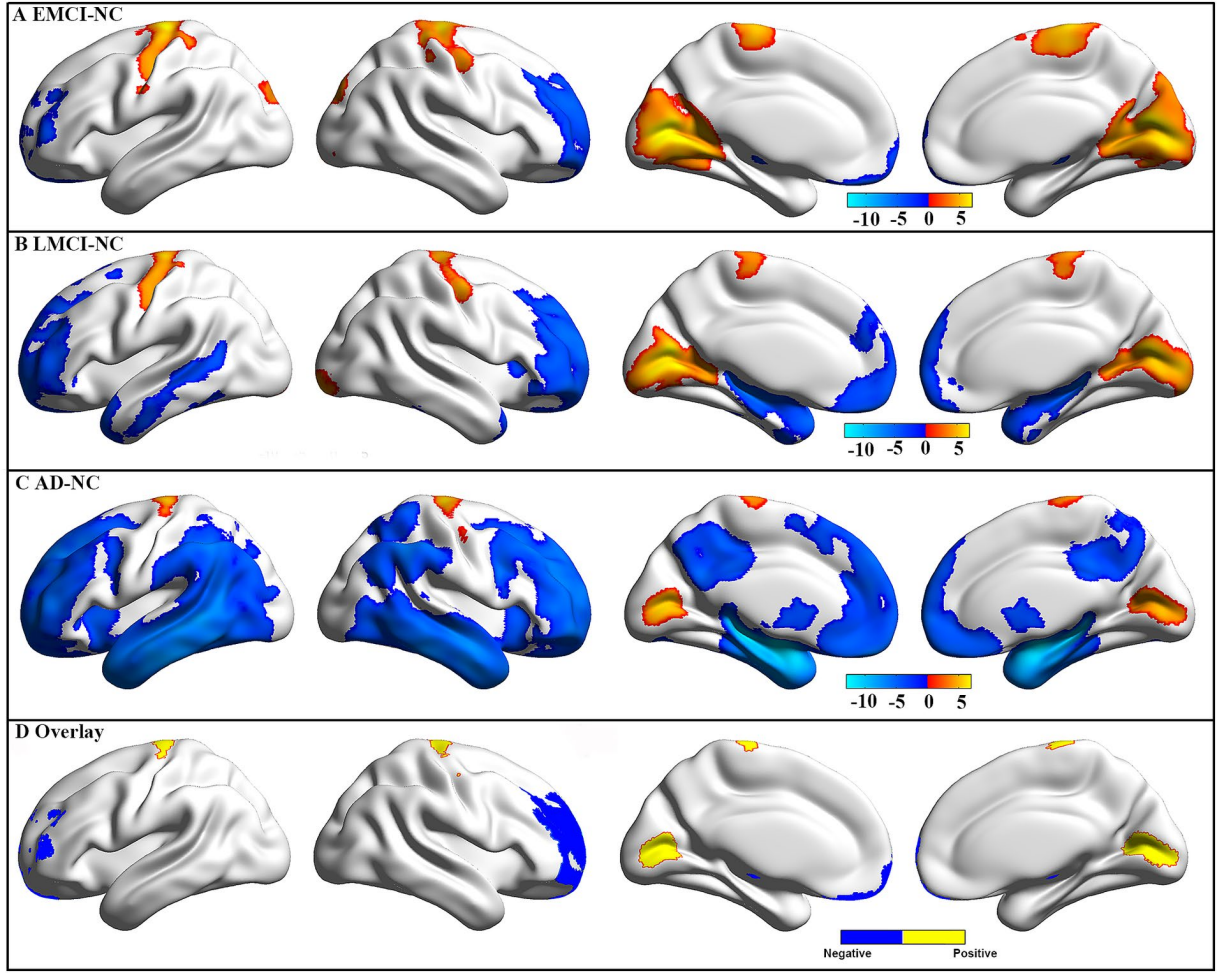
GMV difference between groups. The GMV difference for EMCI (A), LMCI (B), and AD (C) patients as compared to NC. These results were obtained using two-sample t-tests, and multiple comparisons were corrected using a Gaussian random field (GRF, a cluster level of *p* < 0.05 and a voxel level of *p* < 0.001). The color bar represents *t*-values, and a positive *t*-value (warm color) indicates increased GMV in this group compared to NC. Negative and positive (D) overlap among the three groups.

Compared to NC, the EMCI patients showed significantly decreased GMV in the right cerebellum, left rectal gyrus, and bilateral middle frontal gyrus, as well as significantly increased GMV in bilateral calcarine and pre-central gyrus (Figure 2A). Compared to NC, the LMCI patients showed significantly decreased GMV in the right cerebellum and a wide range of regions in frontal, temporal, and subcortical areas, as well as significantly increased GMV in bilateral calcarine and pre-central gyrus (Figure 2B). Compared to NC, the AD patients showed significantly decreased GMV in a wide range of regions in frontal, temporal, parietal, and subcortical areas, as well as significantly increased GMV in bilateral calcarine and pre-central gyrus (Figure 2C). After the intersection, patients showed consistently decreased GMV in the left thalamus, left cerebellum, and bilateral middle frontal gyrus and increased GMV in bilateral calcarine and pre-central gyrus in all three groups (Figure 2D). In addition, a wide range of brain regions showed group differences in GMV among these four groups, which are mainly located in the frontal, temporal, and parietal gyrus (Supplementary Figure S1). Also, the difference in GMV between EMCI, LMCI, and AD was mainly located in the temporal and parietal gyrus (Supplementary Figure S2). Additionally, we found similar changes that showed higher GMV in the right cerebrum and occipital lobe in the men than in the women in each group (Supplementary Figure S3). Therefore, to limit the potential effects of gender on our main results, we used gender as a covariate in our statistical analyses.

### 3.2 AD risk genes associated with GMV differences

**Figure 3:**
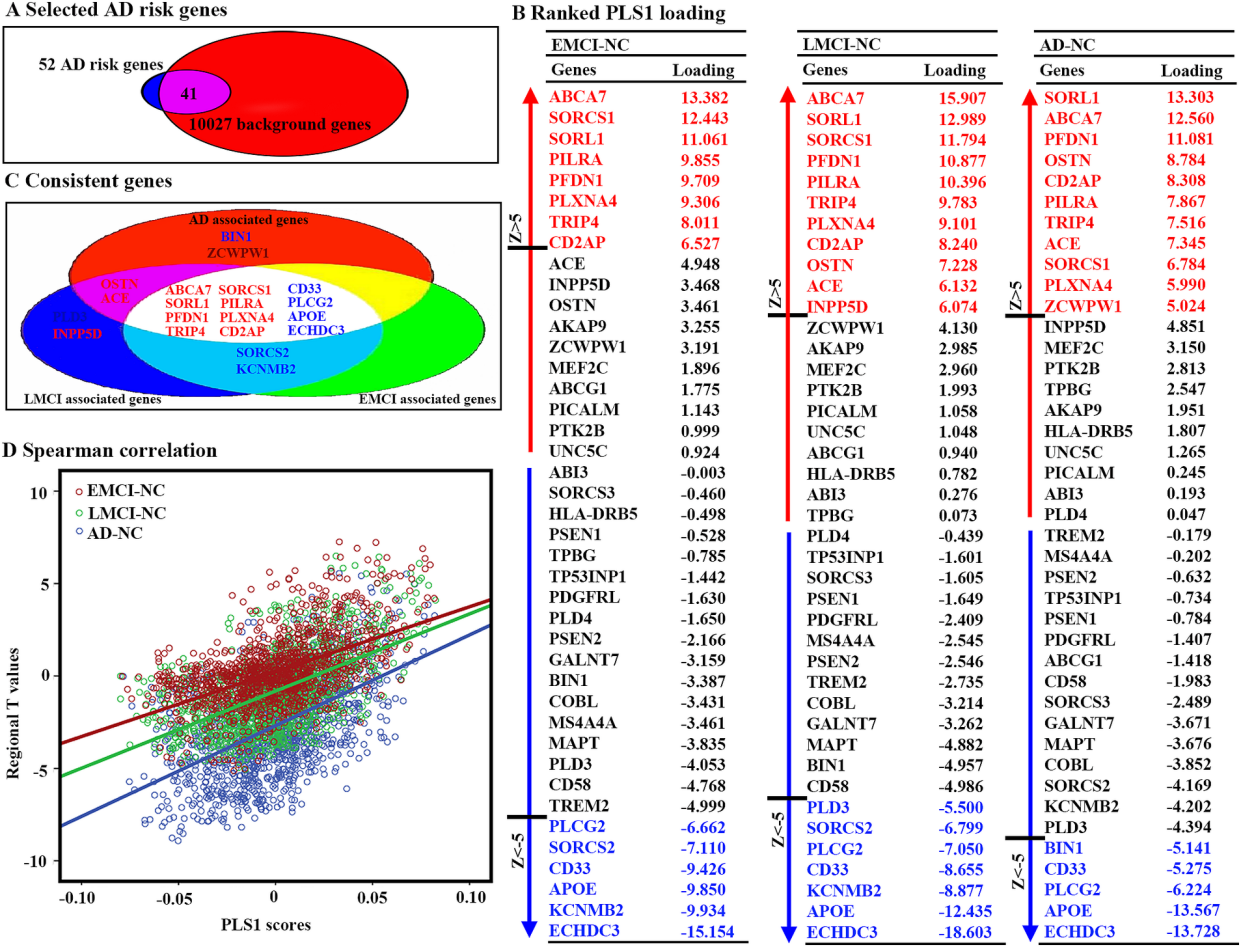
Gene expression level related to regional GMV differences using PLS. (A) Forty-one genes were selected as interested genes. (B) Ranked PLS1 loadings for 41 selected AD risk genes. To estimate the variability of the PLS1 score for each gene, bootstrapping 1,000 times was performed. Z scores were defined as the ratio of the weight of each gene to its bootstrap standard error, and the genes were ranked according to their contributions to PLS1 using univariate one-sample Z tests. The set of genes with *Z* > 5 or *Z* < −5 were considered as positive or negative associated gene lists. All these steps were performed to correct multiple comparisons. (C) Twelve consistent genes among three groups, including eight positively associated genes and four negatively associated genes. (D) Scatterplots of regional PLS1 scores and regional changes of GMV. PLS1: the first component of PLS.

Fifty-two AD risk genes overlapped with 10,027 background genes, resulting in 41 interesting genes for further analyses (Figure 3A). We ranked the normalized weights of PLS1 based on one-sample Z tests for all 41 genes (Figure 3B). We found that eight genes showed a significantly positive association with GMV changes in EMCI patients, 11 genes for LMCI patients, and 11 genes for AD patients, resulting in eight genes after intersection (Figure 3C). Moreover, six genes showed a significantly negative association with GMV changes in EMCI patients, seven genes for LMCI patients, and five genes for AD patients, resulting in four genes after intersection. Notably, we found that the PLS1 weighted gene expression map was spatially correlated with the t-map for EMCI, LMCI, and AD (Figure 3D).

### 3.3 Consistent gene expressions associated with GMV differences

**Figure 4:**
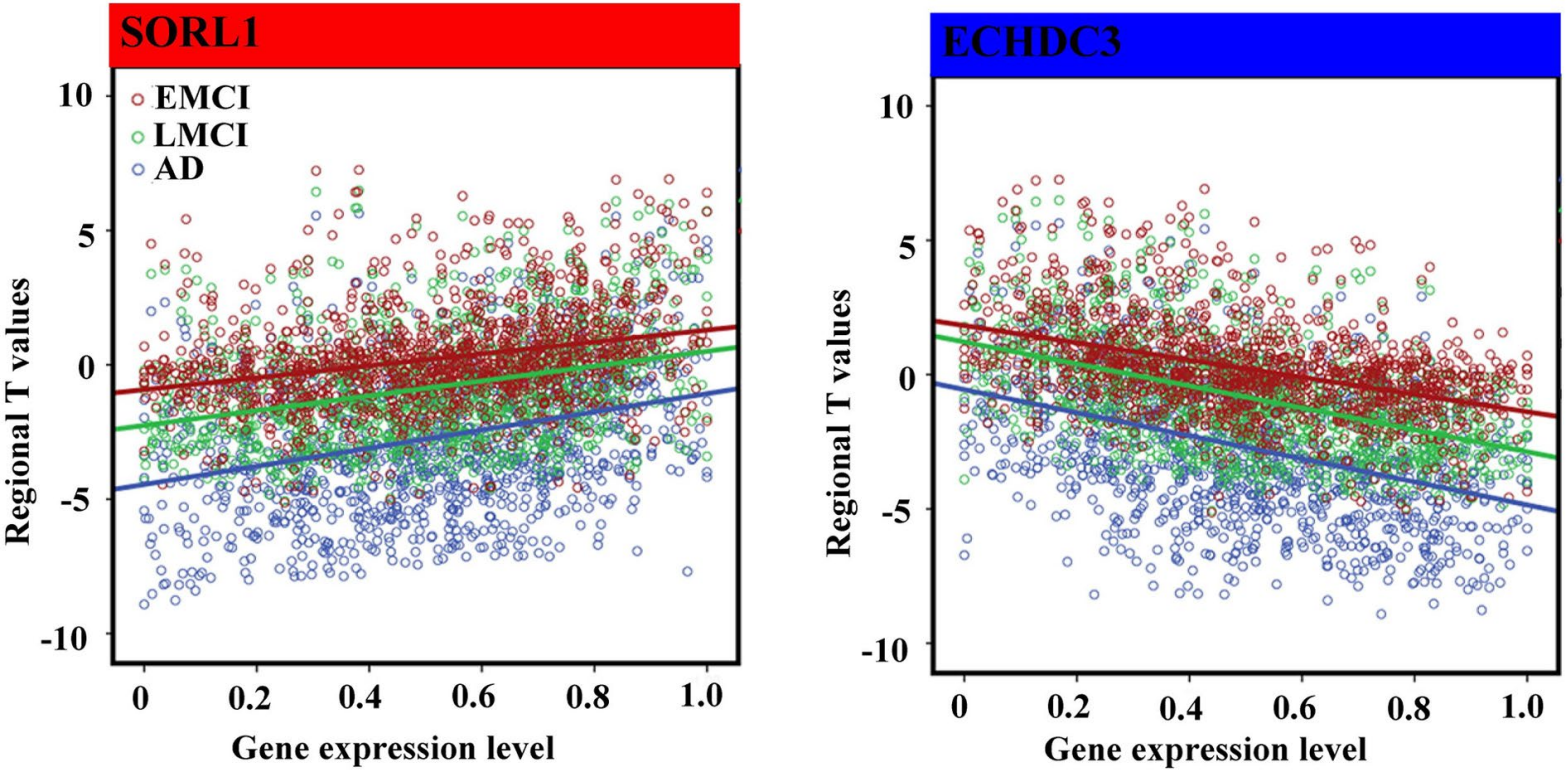
Spearman correlations between gene expression levels of overlap genes and regional *T* values. We performed non-parametric Spearman correlations to explore the relationship between distributions of gene expression level and T values in 1,285 regions in each group. Bonferroni correction (*p* < 4.16 × 10–3 = 0.05/12) was used to correct for multiple comparisons. The blue font represents the negative association, and the red represents the positive association.

For 12 consistent genes, expressions of ABCA7, SORCS1, SORL1, PILRA, PFDN1, PLXNA4, TRIP4, and CD2AP showed significantly positive associations with GMV changes, whereas CD33, PLCG2, APOE, and ECHDC3 showed significantly negative associations (Table 2; Figure 4). Furthermore, we selected one positive and one negative gene from each dataset to present their correlation scatterplots between gene expression values and the t-statistic values of GMV changes.

**Table 2:** Correlation results.

Genes	EMCI-NC (R, P)		LMCI-NC (R, P)		AD-NC (R, P)	
ABCA7	0.280	1.293E-24	0.304	8.03E-29	0.259	4.30E-21
SORCS1	0.295	3.735E-27	0.300	3.21E-28	0.153	3.25E-08
SORL1	0.313	1.183E-30	0.339	7.09E-36	0.311	3.17E-30
PILRA	0.228	1.213E-16	0.219	1.90E-15	0.168	1.43E-09
PFDN1	0.268	1.425E-22	0.304	8.03E-29	0.287	8.70E-26
PLXNA4	0.235	1.388E-17	0.225	2.95E-16	0.143	2.70E-07
TRIP4	0.215	6.034E-15	0.242	1.31E-18	0.192	3.86E-12
CD2AP	0.164	3.405E-09	0.203	2.09E-13	0.210	3.21E-14
CD33	−0.190	6.644E-12	−0.164	3.34E-09	−0.103	2.04E-04
PLCG2	−0.139	6.109E-07	−0.159	9.66E-09	−0.147	1.18E-07
APOE	−0.235	1.386E-17	−0.296	2.55E-27	−0.285	1.64E-25
ECHDC3	−0.425	1.989E-57	−0.499	4.94E-82	−0.403	2.38E-51

### 3.4 Results for sub-groups

The weighted PLS1 scores and r values of Spearman correlation between 12 overlapped genes and GMV differences of cEMCI, cLMCI, sEMCI, and sLMCI compared to NC were significant and highly similar to the main results (Table 3).

**Table 3:** Correlation results for sub-groups.

	cEMCI-NC		sEMCI-NC		cLMCI-NC		sLMCI-NC	
Genes	PLS1	*R* values	PLS1	*R* values	PLS1	*R* values	PLS1	*R* values
ABCA7	12.667	0.239	13.25	0.278	13.799	0.293	13.802	0.298
SORCS1	10.810	0.217	12.566	0.311	11.095	0.262	10.839	0.314
SORL1	8.165	0.240	11.973	0.327	12.868	0.344	13.298	0.301
PILRA	10.854	0.209	9.225	0.220	9.667	0.205	9.878	0.227
PFDN1	6.404	0.184	10.507	0.283	11.070	0.304	11.306	0.279
PLXNA4	7.120	0.195	10.014	0.244	8.459	0.203	8.179	0.234
TRIP4	8.629	0.196	7.551	0.205	8.657	0.223	8.666	0.243
CD2AP	6.386	0.140	6.625	0.165	8.199	0.210	8.752	0.176
CD33	−11.351	−0.206	−7.969	−0.175	−7.383	−0.141	−7.097	−0.188
PLCG2	−7.159	−0.107	−6.644	−0.153	−7.139	−0.159	−6.958	−0.149
APOE	−8.936	−0.185	−9.880	−0.240	−13.004	−0.303	−13.192	−0.270
ECHDC3	−14.868	−0.343	−14.708	−0.437	−15.944	−0.473	−16.488	−0.470

PLS1 scores and Spearman correlations between gene expression levels of overlap genes and regional GMV difference of cEMCI, sEMCI, cLMCI, and sLMCI patients compared to NC. To estimate the variability of the PLS1 score for each gene, bootstrapping 1,000 times was performed. Z scores were defined as the ratio of the weight of each gene to its bootstrap standard error, and the genes were ranked according to their contributions to PLS1 using univariate one-sample Z tests. The set of genes with *Z* > 5 or *Z* < −5 were considered as positive or negative associated gene lists. All these steps were performed to correct multiple comparisons.

### 3.5 Enrichment pathways associated with GMV changes

**Figure 5:**
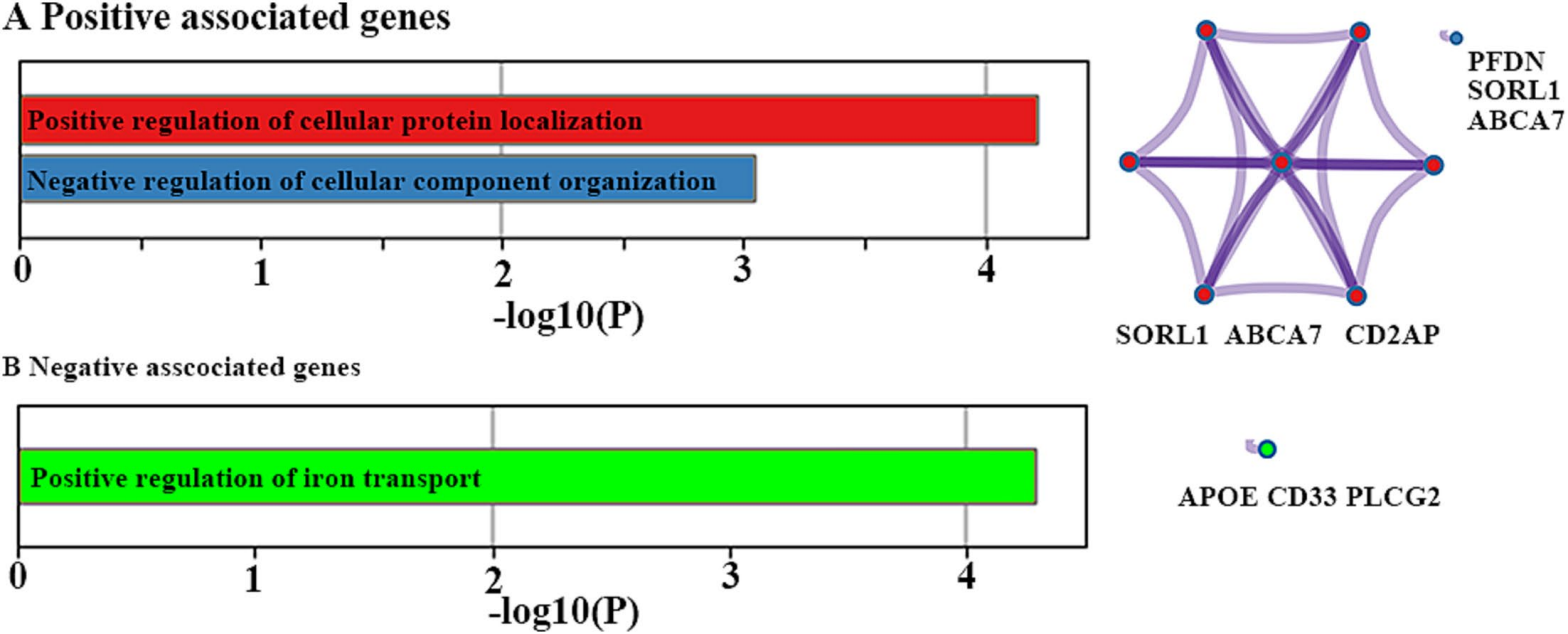
Functional enrichment analyses. Ontology terms and Metascape enrichment network visualization for positive (A) and negative (B) associated genes. The left bar graph represents the set of hallmark genes that we first identified. Accumulative hypergeometric *p*-values and enrichment factors were calculated and used for filtering. The remaining significant terms were then hierarchically clustered into a tree based on Kappa-statistical similarities among their gene memberships. Then, a 0.3 kappa score was applied as the threshold to cast the tree into term clusters. The right-hand network diagram represents the size of the circle represents the number of genes involved in a given term. Each term is represented by a circle node, where its size is proportional to the number of input genes included in that term, and its color represents its cluster identity.

There were two significant GO biological processes, namely positive regulation of cellular protein localization and negative regulation of cellular component organization, for positively associated genes (Figure 5A). There was only one significant GO biological process, namely positive regulation of iron transport, for negatively associated genes (Figure 5B).

## 4 Discussion

Although GWAS, whole-exam sequencing approaches, and meta-analyses showed many AD risk genes, little was known about which genes are associated with GMV changes in the AD spectrum. To narrow this gap, we explored structural changes across the full clinical AD spectrum and performed spatial correlations between these changes and the expression level of AD risk genes. As a result, we identified significant volume atrophy in the left thalamus, left cerebellum, and bilateral middle frontal gyrus across the AD spectrum. These structural changes were consistently associated with expression levels of 12 genes for all three groups and all four sub-groups, which were mainly involved in cellular protein localization, cellular component organization, and regulation of iron transport.

As expected, we identified significant volume atrophy in the left thalamus, left cerebellum, and bilateral middle frontal gyrus across the AD spectrum, suggesting a consistent structural changing pattern. These results could also be used as potential biomarkers for early diagnosis of AD. Moreover, our results were consistent with previous studies reporting GMV reduction in the AD spectrum (Pini et al., 2016; Spulber et al., 2012). A study suggested that GMV reduction might be associated with various micro-structural factors, such as alterations in size, morphology, and number of the cellular and non-cellular components, as well as microglial cells in the cerebral cortex and sub-cortical nuclei (Ji et al., 2021). Indeed, such microstructural changes were widely reported in studies on AD (Davies et al., 2017; Gasparoni et al., 2018; Ishunina et al., 2019; Nicastro et al., 2020). However, the genetic mechanisms resulting in these microstructural changes and GMV reduction remain largely unknown. Moreover, as we know, no direct relation was identified since there is no such large sample of patients with AD who simultaneously have brain-wide transcriptomic and neuroimaging data. Although it is an indirect method of assessing the similarity of spatial distribution patterns between them, our results offered a better understanding of biological mechanisms underlying structural changes in prodromal and clinical AD.

All gene expression values in this study were positive, but the t-statistic values were negative (indicating reduced GMV) or positive (indicating increased GMV). Thus, the negative correlations indicated that brain regions with greater GMV reduction showed higher gene expression, and positive correlations meant that brain regions with greater GMV reduction showed lower gene expression. For instance, we identified the strongest negative association between gene expression of ECHDC3 and GMV changes, whereas the strongest positive association between gene expression of SORL1 and GMV changes across the AD spectrum. The ECHDC3 is a gene that mainly encodes the enzyme enoyl-CoA hydratase domain containing 3, which has been found to be associated with brain neurodegeneration (Tan et al., 2021), especially in AD (Desikan et al., 2015). Moreover, a previous study showed that the pleiotropy at ECHDC3 may be related to the association finding at this locus among persons lacking the APOE ε4 allele (Jun et al., 2017). The SORL1, belonging to both the low-density lipoprotein receptor family and the vacuolar protein sorting-10 domain protein family (Lane et al., 2010), is a key protein involved in the processing of the amyloid-beta (A*β*) precursor protein and the secretion of the Aβ peptide (Campion et al., 2019). It has been observed with a deficiency in the brains of patients suffering from MCI (Sager et al., 2007) and AD (Shen et al., 2014; Dodson et al., 2006) and was supported by results from meta-analyses (Campion et al., 2019). Moreover, the SORL1 genetic variants were reported to modulate or confer the risk of aMCI to probable AD in the Han Chinese population (Chou et al., 2016). Similar to our results, one study also showed that risk variants in SORL1 were associated with less gray-matter tissue in sub-cortical regions, such as the putamen, thalamus, and pallidum (Roshchupkin et al., 2016).

Most importantly, the gene expression level of APOE showed a significantly negative association with GMV changes across the AD spectrum. However, the gene expression levels of APOE sub-types were not available in this study, which mainly included three major polymorphic alleles in humans, namely APOE2, APOE3, and APOE4. Among them, APOE4 remains by far the strongest and most prevalent genetic risk of AD since it has a great influence on two hallmark pathological proteins by modulating the formation of amyloid-β peptide (Aβ) plaques and neurofibrillary tangles containing hyperphosphorylated tau protein (Serrano-Pozo et al., 2021). Similar to our results, many previous studies report significant independent effects of APOE4 genotype on hippocampal volume in MCI and AD (Wang et al., 2015; Saeed et al., 2018; Veldsman et al., 2021; Andrawis et al., 2012), especially in those who progressed to AD (Fang et al., 2019). Other studies described the effects of APOE4 on CA1 (Kerchner et al., 2014), CA3/DG (Mueller and Weiner, 2009), and subiculum (Donix et al., 2010) at sub-regional level. Mean adjusted hippocampal atrophy rates in APOE4 carriers were significantly higher in MCI converter, MCI stable, and AD compared with non-carriers (Manning et al., 2014). In addition to hippocampal atrophy, GMV loss in temporal and parietal lobes, right caudate nucleus, insula, right parietal operculum, the right precuneus, and the cerebellum bilaterally were also reported in APOE4 carriers in patients with MCI (Goni et al., 2013; Spampinato et al., 2011). Moreover, APOE4 has been shown to modify the association between cerebral morphology and cognitive performance in healthy middle-aged individuals (Cacciaglia et al., 2019) and between smaller volumes in the left hippocampus and a tendency to retrieve earlier acquired words in the category fluency task in MCI (Venneri et al., 2011).

In addition to APOE, we also identified that gene expression levels of CD33 and ECHDC3 were negatively associated with GMV changes in MCI and AD. Together, these genes were primarily involved in the positive regulation of iron transport. As we all know, iron is essential for neurons and glia in many aspects (Reinert et al., 2019), such as electron transport, reductase activity of nicotinamide adenine dinucleotide phosphate (NADPH), and myelination of axons. It is available to neurons and glia by transporting from the basolateral membrane of endothelial cells to the cerebral compartment (McCarthy and Kosman, 2015). Although the direct relationship between brain iron and AD remains largely unknown (Tripathi et al., 2017), improper iron transport mechanisms are speculated to lead to the accumulation in various cortical regions and the hippocampus in AD (Mezzaroba et al., 2019; Li et al., 2019). Once exceeding, it might produce reactive oxygen species and pro-inflammatory proteins (Yauger et al., 2019), which cannot be optimally handled in MCI and AD patients (Mills et al., 2010). Indeed, increased iron in the brain has been considered to be one of the primary causes of neuronal death in several neurodegenerative diseases, especially in AD (Lu et al., 2017). In addition, iron is able to induce tau and Aβ aggregation (Sayre et al., 2000) and enhance the toxicity of Aβ (Squitti, 2012). Previous AD models further support the interlinkage between iron metabolism and AD by showing that iron chelators may prevent neuronal loss (Ward et al., 2015). Therefore, it is reasonable to speculate that gray matter atrophy might be related to the dysregulation of iron transport, possibly iron deposit, and neuronal death.

Gene-related changes in GMV were also observed in different brain regions of AD mouse models. For example, a recent study found significant GMV reductions in several brain regions, including the insular cortex (left), basal forebrain (left), subiculum (right), and others, in AAV/APP transgenic mice (Lin et al., 2024). The 3 × Tg-AD mice model, which carries three human mutant genes, has also demonstrated GMV reductions, particularly in the visual cortex (Chiquita et al., 2019). Observations in amyloid beta precursor protein/presenilin 1 (APP/PS1) transgenic AD model mice revealed that the volume of the left hippocampus and right olfactory were reduced (Xu et al., 2024). The APOE gene, which was significantly associated with GMV changes in our study, has also been implicated in exhibiting phenotypes related to cognitive decline in mouse models (González et al., 2023). While the literature predominantly focuses on the APOE gene, the interplay between other genes and GMV in mouse brains remains less explored. Although current literature lacks direct evidence for some genes, we posit that investigating their functions and phenotypes in mouse models could yield significant insights.

Several major methodological limitations are worth mentioning in the current study. First, the gene expression level was calculated from six postmortem brains, whereas the neuroimaging data were obtained from the ADNI dataset. Moreover, we only used gene expression data from the left hemisphere since only two right hemisphere data were available in the AHBA. Therefore, a large sample across the AD spectrum with both brain-wide transcriptomic and neuroimaging data of the same individuals is needed to further verify our results. In future research, we plan to expand the scope of our gene set to include genes from NIAGADS, Alzforum, and ADGC databases, which will increase the likelihood of identifying significant correlations between gene expression and GMV. Second, although most previous studies used surface-based morphology to gray matter segmentation, several previous studies successfully adopted VBM and performed transcriptome-neuroimaging spatial correlation analyses to explore gene expression profiles associated with gray matter volume changes in epilepsy (36444721), children with persistent stuttering (34041495), schizophrenia (37607339), and Alzheimer’s disease (28,105,773, 27,718,423). Due to the algorithm potentially involving brain sites in close spatial proximity but not closely anatomically connected, its potential effects on our results cannot be excluded. Finally, the associations of these structural changes with alterations in the expression of specific genes are indirect and notably weak. Although corrections for multiple comparisons were performed, more attention should be paid to the interpretation of our results.

## 5 Conclusion

In conclusion, this exploratory study linked structural brain changes to gene expression levels by assessing the similarity of spatial distribution patterns. It showed eight genes positively associated and four genes negatively associated with GMV alterations across the AD spectrum, which were validated in four MCI subgroups. These genes were mainly enriched in biological processes related to cellular protein localization, cellular component organization, and iron transport regulation. Collectively, these findings provide a deeper understanding of the biological mechanisms underlying structural changes in both prodromal and clinical Alzheimer’s disease.

## Data Availability

The original contributions presented in the study are included in the article/Supplementary material, further inquiries can be directed to the corresponding authors.

## Footnotes

^1^ http://human.brain-map.org

^2^ https://metascape.org/gp/index.html#/main/step1

^3^ http://adni.loni.usc.edu/

^4^ http://adni.loni.usc.edu/wpcontent/uploads/2010/09/ADNI_GeneralProceduresManual.pdf

^5^ http://adni.loni.usc.edu/methods/documents/

^6^ http://rfmri.org/dpabi

^7^ https://github.com/BMHLab/AHBAprocessing

^8^ https://sourceforge.net/projects/reannotator/
