# Supplementary material for: Consistent genes associated with structural changes in clinical Alzheimer’s disease spectrum
Source: Front Neurosci. 2024 Nov 1;18:1376288. doi: 10.3389/fnins.2024.1376288 (PMC11564164; doi:10.3389/fnins.2024.1376288)
Supplement: Supplementary file 1 [file Data_Sheet_1.pdf]

Supplementary Material

1 Supplementary Figures

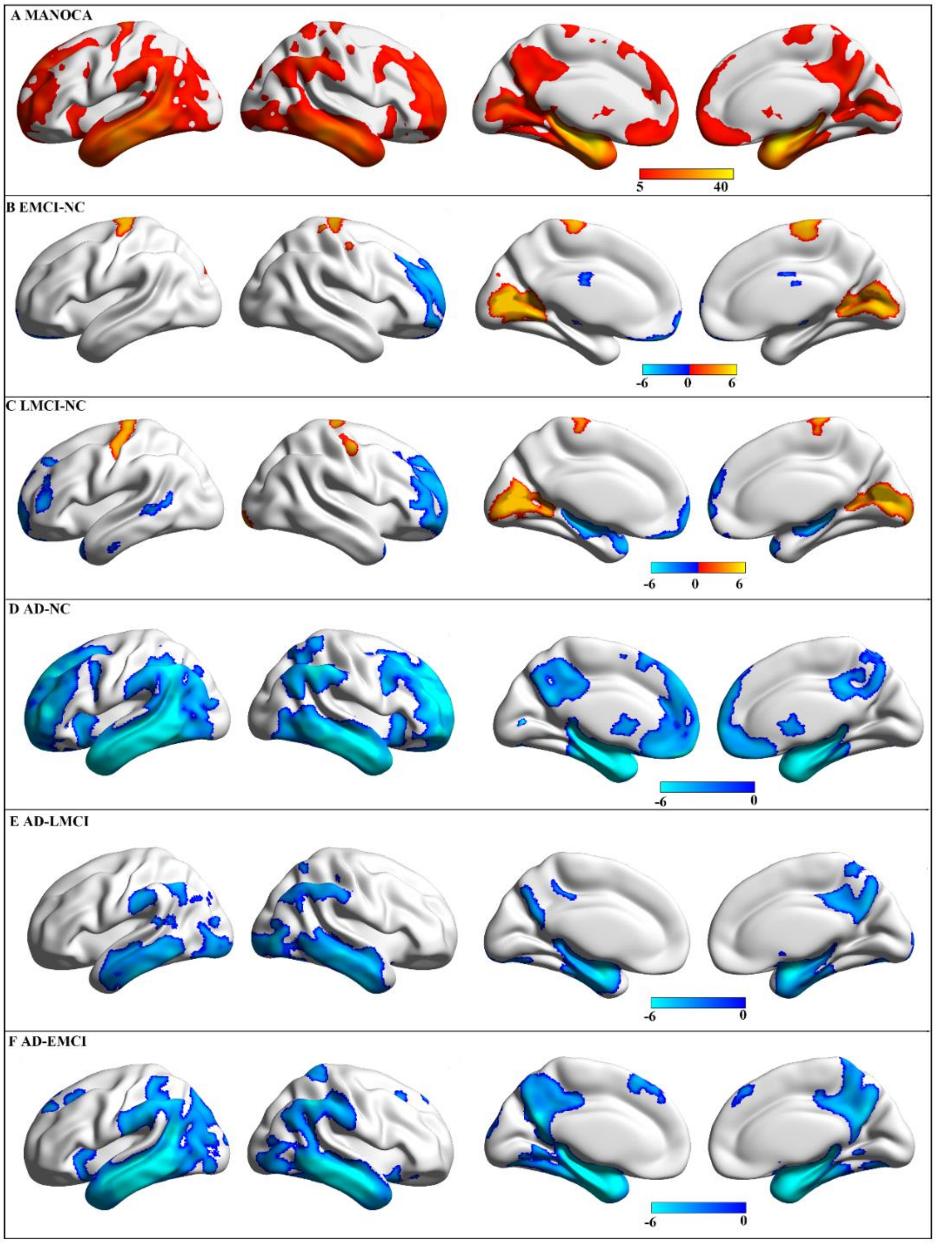

Supplementary Figure S1. The altered gray matter volume among Alzheimer's disease spectrum. The results were obtained by multivariate analysis of variance (MANOVA) among EMCI, LMCI, AD, and NC, and post-hoc analyses between any two groups. The results were corrected using

Gaussian random field (GRF, a cluster level of $p < 0.05$ and a voxel level of $p < 0.001$). There is no significant difference between EMCI and LMCI.

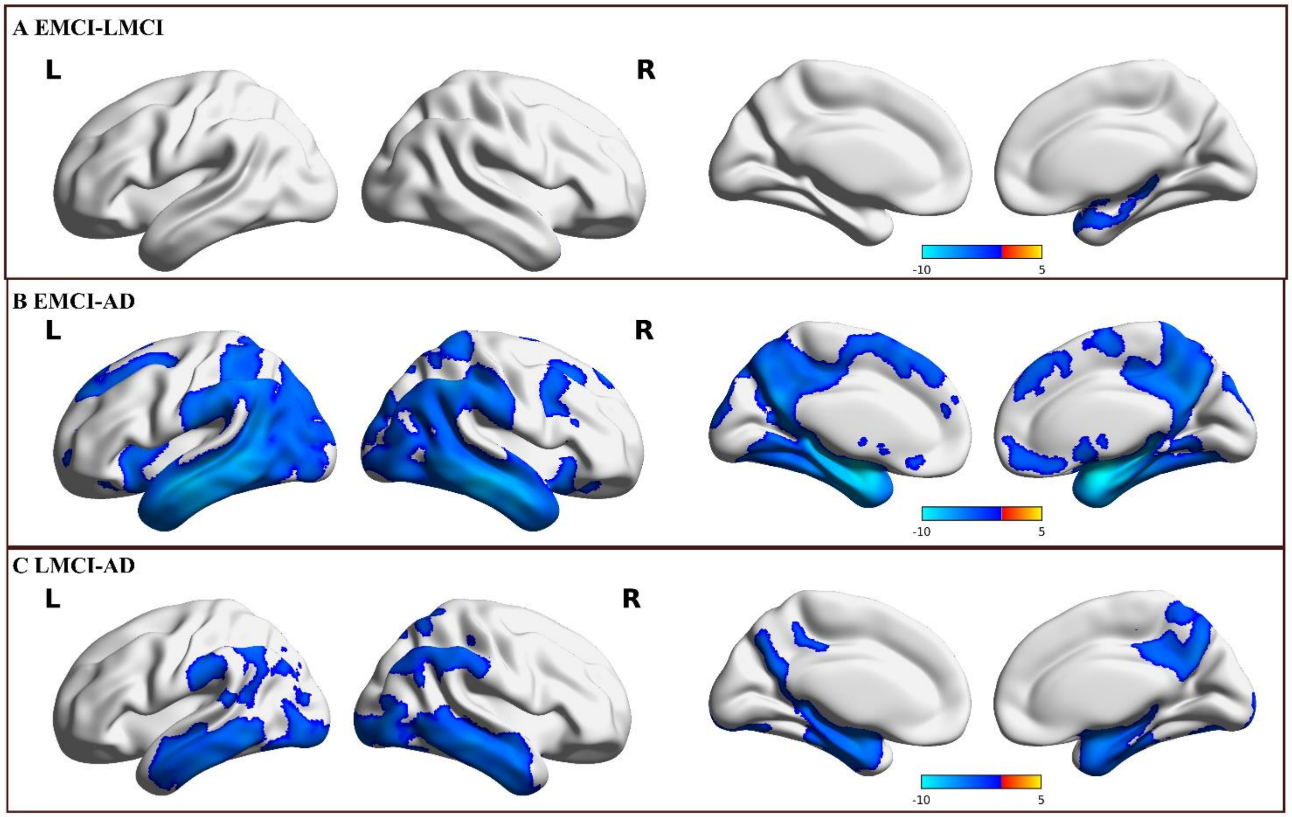

Supplementary Figure S2. The altered gray matter volume among Alzheimer's disease different stages. The results were obtained by two sample t-tests among EMCI, LMCI, and AD. The results

were corrected using Gaussian random field (GRF, a voxel level of $p < 0.001$ and a cluster level of $p < 0.05$).

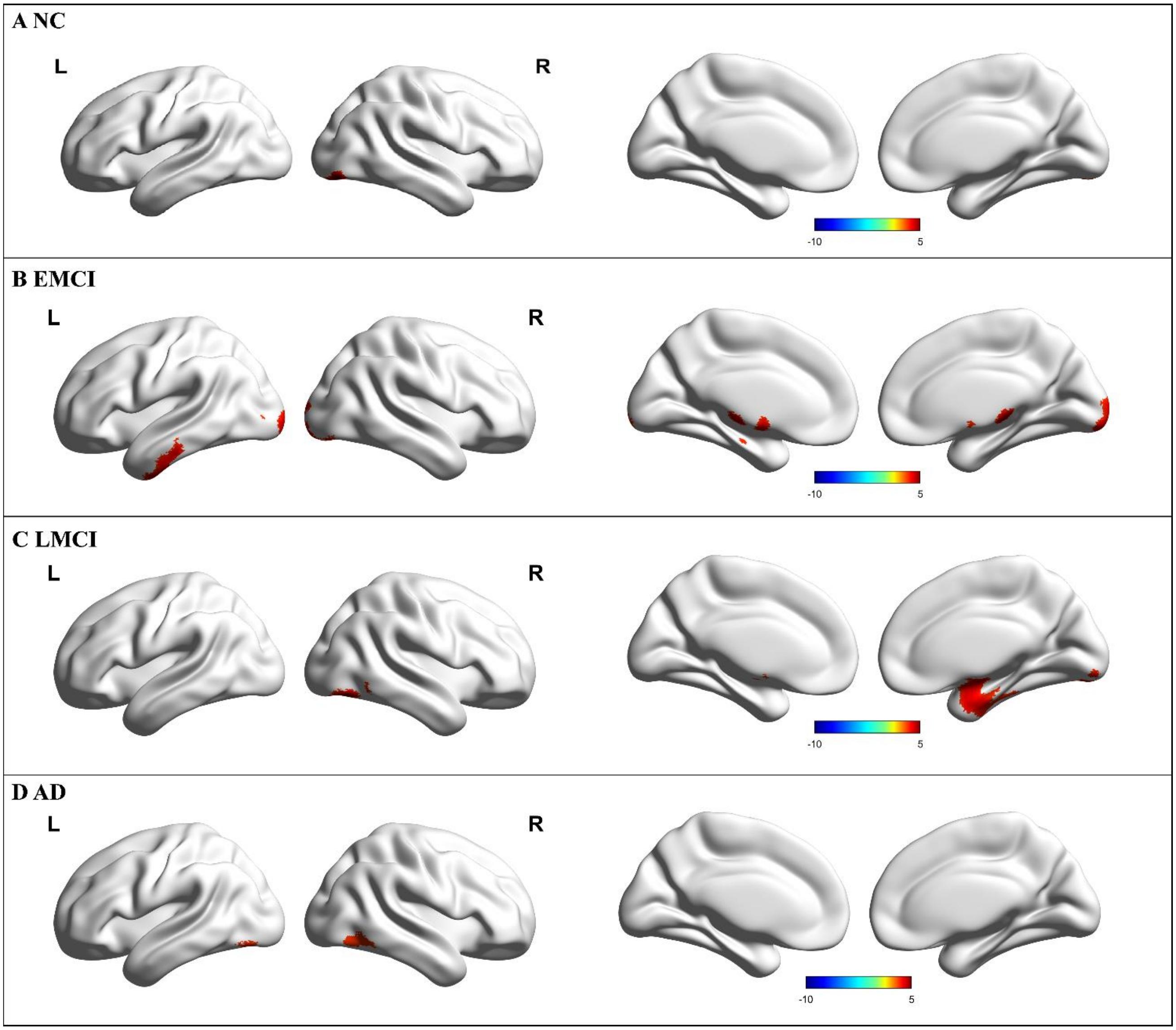

Supplementary Figure S3. The altered gray matter volume between female and male in each group. The results were obtained by two sample t-tests among NC, EMCI, LMCI, and AD. The results were corrected using Gaussian random field (GRF, a voxel level of $p < 0.001$ and a cluster level of $p < 0.05$).
